# A Case of Diabetic Ketoacidosis With Severe Hypertriglyceridemia and Slowly Resolving Metabolic Acidosis

**DOI:** 10.7759/cureus.70228

**Published:** 2024-09-26

**Authors:** Amodini Arora, Owais Ali Khan, Supriya Gupte, Manojkumar G Patil, Shailaja Mane

**Affiliations:** 1 Pediatrics, Dr. D. Y. Patil Medical College, Hospital & Research Centre, Dr. D. Y. Patil Vidyapeeth (Deemed to be University), Pune, IND; 2 Pediatric Endocrinology, Dr. D. Y. Patil Medical College, Hospital & Research Centre, Dr. D. Y. Patil Vidyapeeth (Deemed to be University), Pune, IND

**Keywords:** diabetic ketoacidosis, hypertriglyceridemia, international society for pediatric and adolescent diabetes, triglycerides, type 1 diabetes mellitus

## Abstract

Diabetic ketoacidosis (DKA) is a critical metabolic complication observed in newly diagnosed cases of type 1 diabetes. Severe hypertriglyceridemia, diagnosed in this case, is a rare consequence of DKA. A six-year-old female child presented to the casualty with drowsiness, reduced oral acceptance, difficulty in breathing, hyperglycemia for the past four hours, and polyuria for the past two and a half months. On examination, tachypnea and Kussmaul breathing were present, and the patient was drowsy and confused with a Glasgow coma scale score of 12/15. Grossly lipemic serum was obtained during routine sampling with deranged international normalized ratio (INR), severe hypertriglyceridemia, and refractory metabolic acidosis. According to the International Society for Pediatric and Adolescent Diabetes (ISPAD) guidelines 2023, maintenance fluids were administered intravenously as per blood sugar levels with potassium supplementation, including a deficit of 10%. Insulin was given intravenously at 0.1 IU/kg with a broad-spectrum antibiotic.

## Introduction

Type 1 diabetes mellitus (DM) is a prevalent endocrine and metabolic condition affecting a wide range of juvenile age groups and can result in a variety of metabolic, endocrine, and systemic problems if left untreated. Diabetic ketoacidosis (DKA) is a serious metabolic complication that can occur in up to 40% of newly diagnosed cases of type 1 diabetes [1]. Severe hypertriglyceridemia (HTG) (triglyceride (TG) level > 1,000 mg/dL) is a rare consequence of DKA and has been linked to severe pancreatitis [2]. HTG is defined as a fasting plasma TG content greater than the 95th percentile for age and gender [3]. For children aged zero to nine years, the TG concentration is larger or equal to 100 mg/dL; for those aged 10 to 19, it is greater or equal to 130 mg/dL [4]. Primary HTG is caused by a genetic defect in TG synthesis or metabolism, while secondary HTG frequently results from complications of obesity, poorly controlled DM, metabolic syndrome, and medications such as estrogen [5]. In adults, the interaction of DKA, HTG, and pancreatitis has been addressed. Although 8% of adult DKA cases have severe HTG, there is scant evidence of this combination in children [6]. Therefore, we report a case of HTG in a six-year-old female child having DKA.

## Case presentation

A six-year-old female child presented with drowsiness, reduced oral acceptance, difficulty breathing, and hyperglycemia for the past four hours. The patient has experienced polyuria for the past 2.5 months without evaluation or treatment. She had a mixed diet, receiving 1350 kcal/day and 20 g proteins/day. On examination, she had tachycardia and tachypnea with Kussamaul breathing and was not maintaining saturation on room air. The random blood sugar was 475 mg/dL. On systemic examination, she was drowsy and confused with a Glasgow coma scale (GCS) score of 12/15. A probable diagnosis of DKA was made.

The patient was taken on a high-flow nasal cannula (HFNC) with a flow of 20 L/min and FiO_2_ of 50%. According to the 2023 guidelines of the International Society for Pediatric and Adolescent Diabetes (ISPAD), intravenous 0.9% normal saline bolus at 10 mL/kg was given, and maintenance fluids were administered based on blood sugar levels with potassium supplementation, including a 10% deficit. Insulin was administered intravenously at 0.1 IU/kg, along with a broad-spectrum antibiotic. Subsequently, amlodipine was started due to persistent hypertensive readings.

The routine blood investigations were submitted as presented in Table 1. The venous blood gas revealed metabolic acidosis, and the hemogram showed anemia and leukocytosis. Serum amylase and lipase were elevated. Hyponatremia and hypokalemia were present. The coagulation profile was deranged. Random blood sugar levels and HBA1c were grossly elevated with positive urine ketones.

**Table 1:** Laboratory results during the course of the patient's admission. ALT: alanine transaminase; AST: aspartate transaminase; ALP: alkaline phosphatase; INR: international normalized ratio; aPTT: activated partial thromboplastin time; HbA1c: glycated hemoglobin

Lab Parameter	Value	Reference Range
pH	6.89	7.35-7.45
pCO_2_	11.9 mmHg	35-45 mmHg
pO_2_	72.1 mmHg	75-100 mmHg
HCO_3_	2.2 mEq/L	22-26 mEq/L
Hemoglobin	10.8 g/dL	11.0-14.0 g/dL
Total leukocyte count	12,700/µL	5000-12000/µL
Absolute neutrophil count	8,255/µL	1500-8500/µL
Absolute lymphocyte count	3,683/µL	1500-7000/µL
Platelets	1,43,000/µL	150000-410000/µL
Total bilirubin	0.58mg/dL	0.22-1.20 mg/dL
Direct bilirubin	0.10 mg/dL	Upto 0.5 mg/dL
Indirect bilirubin	0.48 mg/dL	0.1-1.0 mg/dL
AST	27 U/L	8-60 U/L
ALT	21 U/L	7-55 U/L
ALP	194 U/L	142-335 U/L
Total proteins	14.6 g/dL	6.7-8.6 g/dL
Albumin	4.2 g/dL	3.5-5.5 g/dL
Globulin	10.4 g/dL	2-3.5 g/dL
Amylase	3888 U/L	25-115 U/L
Lipase	3154 U/L	8-78 U/L
Prothrombin time	33 seconds	10.83- 13.17 seconds
INR	2.64	0.85-1.15
aPTT	55 seconds	21.75-28.70 seconds
D-dimer	2352 ng/mL	<500 ng/mL
CRP	2.8 mg/dL	<6 mg/dL
Urea	19 mg/dL	17-49 mg/dL
Creatinine	0.4 mg/dL	0.2-0.6 mg/dL
Sodium	124 mmol/Lt	138-145 mmol/Lt
Potassium	3.3 mmol/Lt	3.5-5.1 mmol/Lt
Chloride	101 mmol/Lt	98-107 mmol/Lt
Random blood sugar level	444 mg/dL	<140 mg/dl
HbA1c	16.2	<5.7%
Urine ketones	4+	Negative
USG abdomen	No abnormality detected.	

**Figure 1:**
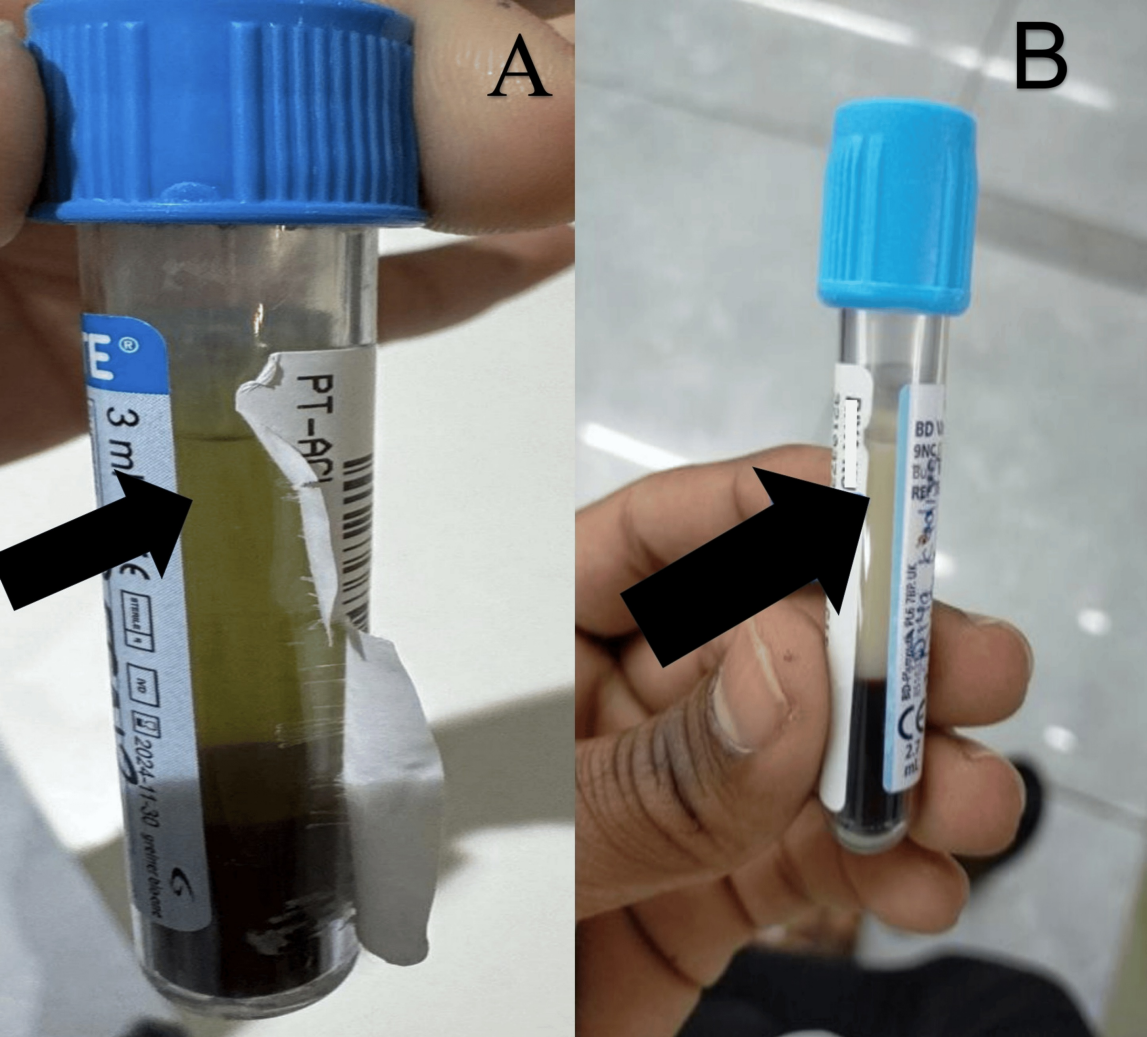
(A) The normal appearance of the serum and (B) the grossly lipemic serum after centrifugation of a venous blood sample collected in a sodium citrate coagulation vial.

A difficulty was encountered during the sample collection due to the grossly milky outflow. The central clinical laboratory struggled to process the sample as it would turn milky immediately. Following several centrifugation cycles, the serum exhibited significant turbidity and lipemia, as illustrated in Figure 1.

Hence, a fasting lipid profile was sent, and HTG was reported (triglyceride level of 2488 mg/dL). The intravenous insulin drip was continued and then shifted to subcutaneous insulin after her appetite improved. Hyperglycemia and metabolic acidosis were initially refractory to correction and required insulin at 0.2 IU/kg until they resolved. Raised lipase levels and amylase levels were also reported in the patient. Her serum amylase, lipase, and TG levels started trending down approximately seven days post-admission and therapy. A variation in the TG values was noted over three weeks, as seen in Table 2.

**Table 2:** Serial variation in serum triglyceride values. HDL: high-density lipoprotein; LDL: low-density lipoprotein; VLDL: very-low-density lipoprotein

Lipid Profile	Reference Range	On the Day of Admission	After 7 Days of Admission	On 3 Weeks Follow-up
Total cholesterol	<200 mg/dL	309 mg/dL	205 mg/dL	177 mg/dL
Triglyceride	<150 mg/dL	2488 mg/dL	207 mg/dL	177 mg/dL
HDL	>50 mg/dL	120 mg/dL	18 mg/dL	12 mg/dL
VLDL	<30 mg/dL	498 mg/dL	40 mg/dL	33 mg/dL
LDL	<100 mg/dL	300 mg/dL	160 mg/dL	124 mg/dL
Total cholesterol:HDL ratio	<4.5	30.90	11.60	9.38

Ultrasonography of the abdomen was done to rule out acute pancreatitis and was within normal limits. A rare complication of type 1 DM and DKA was hence diagnosed. A resolution in metabolic acidosis and abnormal GCS was observed after fluid supplementation, BSL and urine ketone monitoring, and insulin infusion. At the three-week follow-up, there was adequate weight gain, improved oral intake, and blood sugar level control without secondary opportunistic infections, allowing the resumption of routine activities.

## Discussion

DKA, a frequent consequence of type 1 DM, is a life-threatening condition in which insulin deficiency stimulates lipolysis in adipose tissue, increasing free fatty acids and promoting the synthesis of very-low-density lipoprotein (VLDL) in the liver [7]. The decrease in lipoprotein lipase activity in peripheral tissue reduces VLDL clearance from plasma, resulting in HTG [8].

Our patient presented with life-threatening DKA and severe HTG, which was treated with fluid resuscitation and insulin infusion. A similar case was reported, which was managed by administering insulin, omega oil, and fibrates and maintaining hydration, and could lower the TG levels to normal in five days [9]. TGs are transported through the circulation pathophysiologically by specific lipoproteins secreted from the small intestine and digested in the peripheral tissues. The subsequent process is lipolysis, which results in the release of fatty acids. This causes hyperviscosity, probable acidosis, and ischemia in the pancreatic capillary beds [10]. In response, the ischemia and inflammation stimulate the pancreas to secrete a large quantity of amylase and lipase. TG levels of more than 500 mg/dL elevate the risk of developing pancreatitis, forming a vicious triad. Hence, the recommendation is that all three factors must be treated simultaneously via good hydration, insulin, and targeted reduction in TG levels through lifestyle changes and drugs such as statins and fibrates.

As potassium is driven into the intracellular compartment during the acute therapy of DKA, the serum potassium level may drop abruptly. The serum potassium levels must be monitored every one to two hours in the first five hours post-commencement of treatment since major changes occur within the same period. In our case, potassium replacement was commenced accordingly. Patients diagnosed with DKA with HTG should undergo genetic testing to rule out the possibility of familial causes and concurrent mutations in the *LPL* gene, as this has been hypothesized to be necessary. In DKA patients, a mutation in the *LPL* gene can be a causative agent for HTG that is severe, long-lasting, and non-resolving solely on insulin therapy. An abnormality in the lipoprotein co-factors might be the root cause of severe HTG that does not last for an extended period. It is possible that under normal circumstances, a slight aberration in lipid metabolism may lead to the development of hyperlipidemia in response to stress caused by illnesses such as diabetes. In the present case, HTG was managed with insulin alone, without the use of lipid-lowering drugs. However, in severe HTG, some physicians consider plasma exchange to prevent complications. There is a possibility of electrolyte imbalance if serum TG concentration surpasses 2,500 mg/dL due to intracellular migration of serum lipid components. In our case, pseudo-hyponatremia was observed, and overtreatment with hypertonic saline was avoided.

## Conclusions

Severe HTG is an unusual and critical outcome of DKA. Clinicians must be mindful of the potential for HTG in patients with DKA and acute pancreatitis, and this triad should be promptly recognized for appropriate management. However, this triad is relatively common in adults but rare and poorly documented in the pediatric age group. In our case, the patient responded to intravenous fluid therapy and insulin infusion with a decrease in TG levels. In DKA, a low-lipid diabetic diet is essential for TG control. Such cases must be reported to provide more data for future research.
